# Dataset: percent of population covered by local government mask orders in the US

**DOI:** 10.12688/f1000research.26967.1

**Published:** 2020-10-22

**Authors:** Philip Jacobs, Arvi Ohinmaa

**Affiliations:** 1 Department of Medicine, University of Alberta, Edmonton, Alberta, T5T 2W8, Canada

**Keywords:** COVID-19, Mask orders, Local government, Cities, Counties

## Abstract

We present a dataset covering the extent of local mask orders between April and August 2020, in states which did not have statewide orders (and hence 100% coverage).  We obtained data from national and regional newspaper and broadcaster web-based articles, and city and county web pages. The information that we abstracted included: city or county of ordinance, date that the ordinance took effect, and the population of the city or county. In 14 states, city or county governments issued mask-wearing orders, and from our dataset it can been seen that the median population covered in the states was 37.5%; the coverage ranged from 1.6% (New Hampshire) to 77.1% (Arizona).  The dataset can be accessed from: https://doi.org/10.7939/DVN/A9C1UU

## Introduction

By August 9, 2020, governors in 35 US states had issued statewide mandates for persons to wear COVID-19 protective face masks ^1^. These mandates ensured that the entire state populations were covered, although population adherences to the mandates were not complete ^1^. In 15 of the remaining 16 states, until September 9, some local governments (county or municipal) had also issued mandates for persons to wear masks. Mask wearing in public has become a bulwark against COVID-19, and it is desirable to determine the population proportions in the states that are only covered by local ordinances. We present a dataset that provides this information.

## Methods

**Figure 1.**
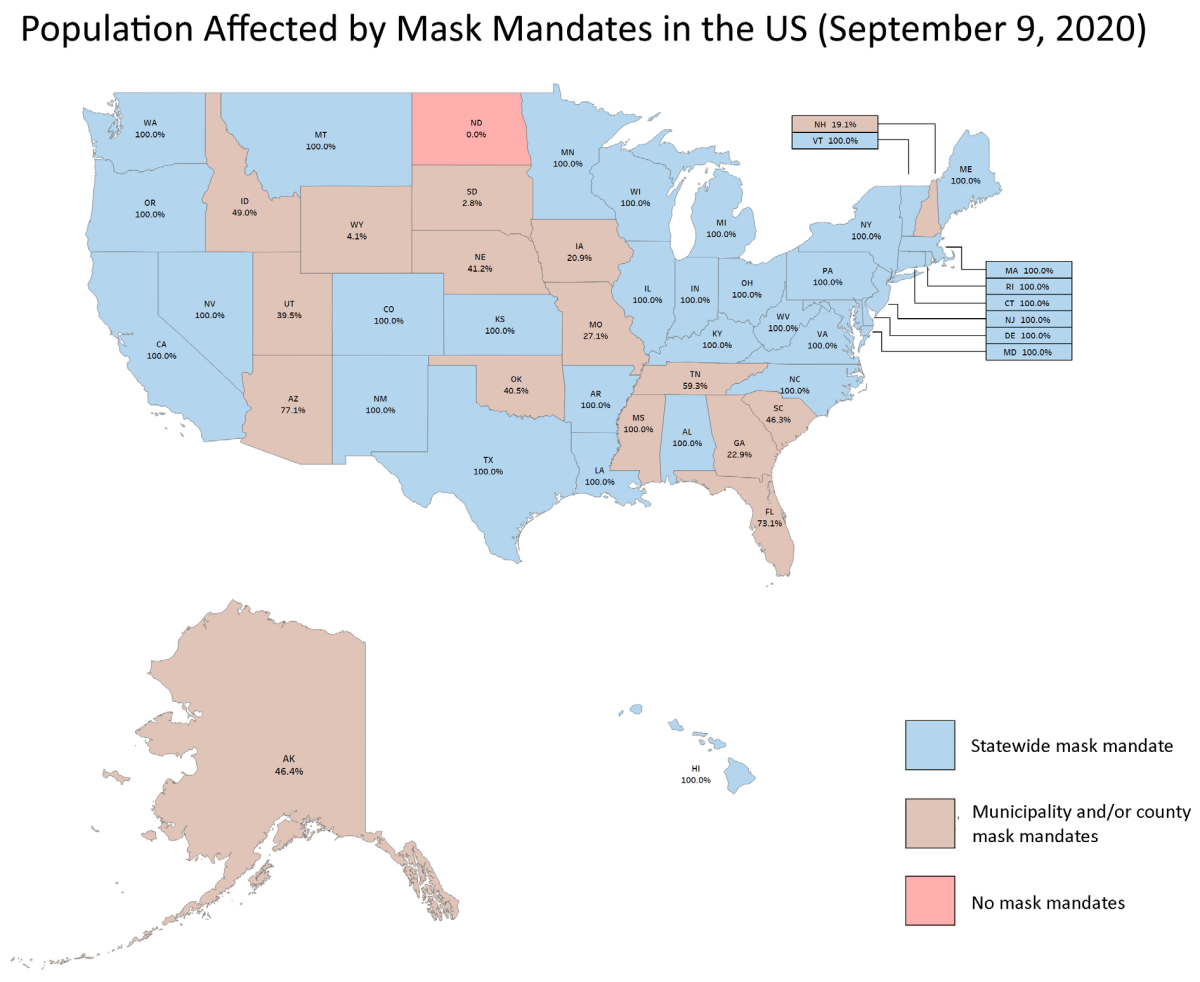
Number of local governments issuing mask mandates. The number of mandates ordered by separate city or county governments in each of 14 states which did not have a central mandate. Blue indicates cities ordering a mandate, brown indicates counties and black indicates a tribal council.

Starting with the 14 states with only local mandates (see Figure 1), we searched for lists of counties and towns or cities that introduced mask orders effective September 9 or earlier. We conducted a Google search for each state with the combined terms “State name” (e.g., Florida), “COVID-19”, “mask” or “face mask”, and “county order” or “city order,” Usually, we found at least one article with a mention of counties or cities with face masks. In some states the list was large; in those states we searched for internet articles with complete lists; we found lists for Arizona ^a^, Florida ^b^, South Carolina ^c^, Tennessee ^d^, and Wisconsin ^e^. We then searched Google for newspaper or broadcast sites that covered the mask order for each county or city using “State name” (e.g., South Carolina), “city name” (e.g.,” Columbia”), or “county name” (e.g., “Richland County”), “COVID-19”, and “mask order”. We also searched for the ordinances on the city, town, or county government web pages.

We collected the following data: the state and city or county name; the date each order became effective; the 2019 population of each relevant city or town ^2^ and county ^3^. We used these populations as estimates of the number of people in each area who were under mask orders. If both city or town and county had issued mask orders, we used only the county population as our measure of the number of people covered. 

## Dataset description

In the accompanying Excel file ( *Underlying data*), in the column ‘NAME’ we list the counties, cities and towns with mask orders. We identify cities or towns with an “M” (municipality) and counties with a “C.” We also show the date that each order came into effect. We embed the internet address of the related newspaper or broadcast article in the “Date in effect” column. We also record the population of each city, county and state. If both a city and its county had a mask order, we used the county population as our indicator of coverage. We recorded the city population in a separate column. 

**Figure 2.**
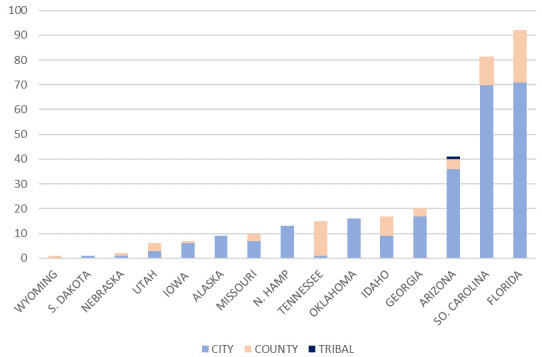
Percent of population by US state under mask orders. The figure shows the percent of each state’s population that was under a local or statewide mask order by September 9, 2020.

**Figure 3.**
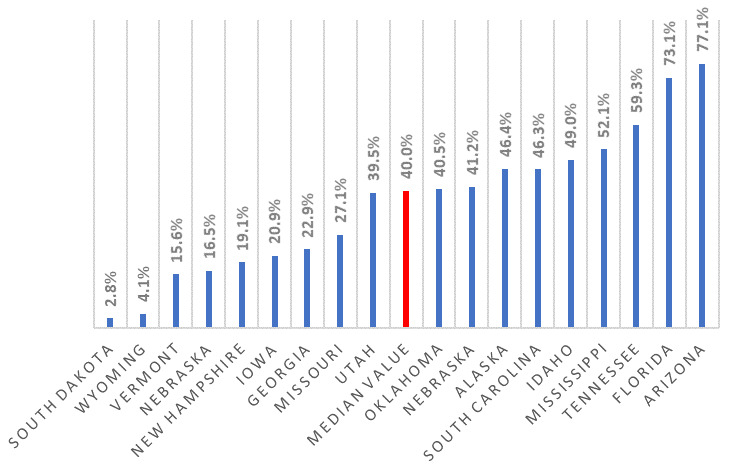
Proportion of state populations covered by local mask orders. The average coverage for all 14 states, which have only local coverage, is 37.5%.

Data on the number of ordinances for cities and counties in each relevant state is shown in Figure 2. Counties took the initiative in Wyoming, Nebraska, Utah, and Tennessee. In states with more orders, including Arizona, South Carolina and Florida, cities took the initiative. In Arizona there was also an order from a Tribal Council. In Figure 1, we show the country map, identifying states with statewide orders (blue), local government only orders (brown), and no orders (light red). In this figure, we also present the percent of each state’s population that was covered by mask orders. For the states with statewide orders, coverage is complete (100%). One state, North Dakota, did not have any orders up until our cutoff date of September 9 (in South Dakota, one city, Brookings, enacted an order on September 9). In Figure 3, we show the ranking of states by percent population covered, for states with only local mandates.

## Summary

Our dataset shows the population coverage for mask mandates in states where local governments took policy initiatives. Coverage in these states varied widely and is an important component of any analysis of COVID-19 prevention policies.

There is little nationwide information available on the degree of coverage in states with local mandates. There is no central body that collects and organizes this data and makes it publicly available. This dataset addresses that deficiency. However, there are limitations in collecting this information. Firstly, mask order enactment dates keep changing and local governments keep adding or terminating enactments as the local COVID-19 situation changes. Secondly, news bureaus do not always provide the current situation. Finally, data on county and city orders are not always kept in a central place for public information.

## Data availability

University of Alberta Library Dataverse: Mask Orders: Local Government, https://doi.org/10.7939/DVN/A9C1UU ^4^.

Database contains detailed collected data for 15 states with local orders and more general data for 34 states with statewide orders:

- **Part 1. Detailed data**
  - A. State
  - B. Location
  - C. Location’s designation: Municipality or County
  - D. Date order became in effect + source data (embedded)
  - E. Population that is contributed to the state population measure
  - F. Actual population (some double counting)
  - G. Blank
- **Part 2 State-level data**
  - H. State
  - I. State population
  - J. Population under mask orders in state
  - K. Per cent of population under mask orders in state
  - L. Number of municipalities with orders
  - M. Number of counties with orders.

Data are available under the terms of the Creative Commons Zero "No rights reserved" data waiver (CC0 1.0 Public domain dedication). 

## Notes

^a^ Source: https://floridapolitics.com/archives/342364-beyond-the-veil-what-face-mask-requirements-are-in-place-in-florida

^b^ Source: https://www.tennessean.com/story/news/2020/07/06/does-your-tennessee-county-require-face-masks-worn-public/5387850002/

^c^ Sources: https://ktar.com/story/3298944/heres-where-arizona-cities-stand-on-requiring-face-masks/; https://www.fox6now.com/news/list-wisconsin-cities-with-mask-mandates

^d^ Source: https://www.thestate.com/news/coronavirus/article244628692.html

^e^ Source: https://www.wistv.com/2020/07/08/full-list-face-mask-ordinances-place-across-sc/
