# Supplementary material for: Dataset: percent of population covered by local government mask orders in the US
Source: F1000Res. 2020 Oct 22;9:1267. [Version 1] doi: 10.12688/f1000research.26967.1 (PMC7871414; doi:10.12688/f1000research.26967.1)
Supplement: Peer review history

# Peer review: Reviewer response for version 1

**DOI:** 10.5256/f1000research.29788.r77749

**Published:** 2021-02-08

**Authors:** Jonathan Harris

**Affiliations:** 1 National Association of Counties, Washington, DC, USA

In this report, the authors created a new, useful dataset on mask mandates across the country and the share of the population these mandates cover in each state. Since no centralized database is available for these policies, the researchers used sound methodology to create the dataset through targeted Google searched.

 The authors covered the primary limitations of their methodology, including (1) the difficulty it will be to keep this dataset updated with ever-shifting policies, (2) having to rely on news outlets for information and (3) the lack of a centralized database for this information. On the other hand, this dataset will be easy to check, since it is all publicly available.

Are sufficient details of methods and materials provided to allow replication by others?

Yes

Is the rationale for creating the dataset(s) clearly described?

Yes

Are the datasets clearly presented in a useable and accessible format?

Yes

Are the protocols appropriate and is the work technically sound?

Yes

Reviewer Expertise:

County government and related areas

I confirm that I have read this submission and believe that I have an appropriate level of expertise to confirm that it is of an acceptable scientific standard.

# Peer review: Reviewer response for version 1

**DOI:** 10.5256/f1000research.29788.r73597

**Published:** 2021-01-04

**Authors:** Mayvis Rebeira

**Affiliations:** 1 Canadian Health Economics Centre, University of Toronto, Toronto, ON, Canada

The dataset provides information on the proportion of the population covered by mask mandates in states where local governments had taken policy initiatives. The period of the data collection is between April and August 2020. Data was obtained from national and regional newspaper and broadcaster web-based articles, and city and county web pages.

 The data includes two parts: Part 1 contains municipal/county data and date the mask order was in effect and the population. The source data does not seem easily accessible here. Part 2 contains data on states where there was state-wide enactment of masks and the number of counties/municipality with local ordinances.

 Based on these data, the authors found that in 14 states, city or county governments issued mask-wearing orders, and from the dataset, the authors conclude that the median population covered by mask enactments in the states was 37.5% and the coverage ranged widely from 1.6% in New Hampshire to 77.1% in Arizona.

 The method used to derive the dataset from public sources is explained including the search terms.

 The authors noted several important limitations to the dataset including the fact that mask order enactment dates keep changing and local governments keep adding or terminating enactments. Further, the authors rely on publicly available sources for the information as not all news bureaus may have provided such information. As such there may be data that is missing and further validation and update of the dataset may need to be conducted at a later stage when used to analyze COVID-19 policies. Since the data is publicly available, the verification of data can be conducted and further additional data can be added as more publicly information on mask ordinances become available publicly.

Are sufficient details of methods and materials provided to allow replication by others?

Yes

Is the rationale for creating the dataset(s) clearly described?

Yes

Are the datasets clearly presented in a useable and accessible format?

Yes

Are the protocols appropriate and is the work technically sound?

Yes

Reviewer Expertise:

Health Economics, Econometrics, Disease Risk Factor, Health Policy

I confirm that I have read this submission and believe that I have an appropriate level of expertise to confirm that it is of an acceptable scientific standard.
